# GCMM: graph convolution network based on multimodal attention mechanism for drug repurposing

**DOI:** 10.1186/s12859-022-04911-8

**Published:** 2022-09-13

**Authors:** Fan Zhang, Wei Hu, Yirong Liu

**Affiliations:** School of Information Science and Technology, University of Science and Technology of China, Hefei, China

**Keywords:** Computational drug repurposing, Graph convolutional network, Attention mechanism, Heterogeneous information

## Abstract

**Background:**

The main focus of in silico drug repurposing, which is a promising area for using artificial intelligence in drug discovery, is the prediction of drug–disease relationships. Although many computational models have been proposed recently, it is still difficult to reliably predict drug–disease associations from a variety of sources of data.

**Results:**

In order to identify potential drug–disease associations, this paper introduces a novel end-to-end model called Graph convolution network based on a multimodal attention mechanism (GCMM). In particular, GCMM incorporates known drug–disease relations, drug–drug chemical similarity, drug–drug therapeutic similarity, disease–disease semantic similarity, and disease–disease target-based similarity into a heterogeneous network. A Graph Convolution Network encoder is used to learn how diseases and drugs are embedded in various perspectives. Additionally, GCMM can enhance performance by applying a multimodal attention layer to assign various levels of value to various features and the inputting of multi-source information.

**Conclusion:**

5 fold cross-validation evaluations show that the GCMM outperforms four recently proposed deep-learning models on the majority of the criteria. It shows that GCMM can predict drug–disease relationships reliably and suggests improvement in the desired metrics. Hyper-parameter analysis and exploratory ablation experiments are also provided to demonstrate the necessity of each module of the model and the highest possible level of prediction performance. Additionally, a case study on Alzheimer’s disease (AD). Four of the five medications indicated by GCMM to have the highest potential correlation coefficient with AD have been demonstrated through literature or experimental research, demonstrating the viability of GCMM. All of these results imply that GCMM can provide a strong and effective tool for drug development and repositioning.

## Introduction

The creation of new drugs still takes a long time, despite technological advancements and rising investment in this area [1]. The minimal number of brand-new medications that have been authorized for sale in recent years cannot meet the healthcare needs of the modern world [2]. DR research has emerged as a potential area in drug discovery and is attracting more interest [3] in order to increase the effectiveness and dependability of medications. There are numerous examples of drug repurposing that have been effective in finding new uses for already-approved medications. The pharmaceutical business can use two methodologies, known as in-silico DR and activity-based DR, respectively [4, 5]. Activity-based DR is frequently experimental and time-consuming [6]. A significant amount of biological data is being generated for the expertise repositioning process at a lower cost thanks to the quick development of biomedical technologies, such as high-throughput screening [7] and next-generation sequencing technology [8]. Since the repositioning medicine has successfully completed three stages of clinical testing and prior information may be questioned [9], computational DR is far less expensive and more accessible than experimental techniques [10].

Feature-matching-based and molecular docking techniques are two examples of traditional computational DR methods [11]. It has become increasingly and successfully possible to predict the links between drugs and diseases and between drugs and proteins thanks to the development of artificial intelligence technology [12]. As a result, algorithms have been developed that can anticipate how certain drugs would interact with certain diseases or other organisms, and their performance is steadily getting better. The similarity-based algorithm is based on the idea of guilt by association [13], which is the fundamental idea in the field of DR. According to the theory of guilt by association, the likelihood that two drugs will be associated with the same disorders is increased in direct proportion to how functionally similar they are [14].

Prior research in DR has mostly concentrated on machine learning algorithms. Laplacian regularized least square (LapRLS), a semi-supervised learning technique used to predict drug-protein interactions, was proposed by Xia et al. [15]. Bayesian ANalysis to Determine Drug Interaction Targets (BANDIT4F), created by Madhukar et al. [16], enables precise prediction of drug interactions with particular targets, including identifying particular targets for a wide range of small molecules and various modes of action on the same target. However, the majority of machine learning methods largely rely on feature engineering and expert knowledge. As an extension of the artificial neural network, deep learning [17] is also widely used in computational drug repurposing. The advantage of deep learning is that it can learn the complex relationship between input features and output decisions from large-scale data. To learn drug feature representation, Zeng et al. [18] constructed multiple drug-related networks and integrated them with a multi-modality autoencoder named DeepDR. Then, by feeding the known drug features and drug–disease correlations into the variable differential autoencoder’s pre-training, the prospective drug–disease associations are anticipated. When DeepDR’s results are evaluated using cross-validation and case studies, they outperform traditional methods in identifying novel drug–disease connections. The relationships between drugs and diseases can be thought of as a bipartite graph, which can be thought of as a heterogeneous biological network made up of relationships between drugs, diseases, and drug targets. As a result, the graph embedding approach, particularly the graph neural network method [19], is gradually applied to this issue. In order to anticipate probable drug-target interactions, Wan et al. [20] developed a neural integration of neighbor information from an HN (NeoDTI). NeoDTI automatically learns topology-preserving representations while integrating a variety of data from HN. In order to aggregate the embeddings from several graph convolution layers using an attention mechanism, Yu et al. [21] suggested a layer attention graph convolutional network (LAGCN) for the prediction of drug–disease associations. Li et al. [36] established the NIMGCN, which applies GCN to the networks for miRNA similarity and disease similarity, respectively, and adds a neural inductive matrix completion to predict the relationships between miRNA and diseases.

Although computational DR performance for existing techniques has been remarkable, there are still several limitations. Some strategies initially simply take into account comparable drug information while ignoring the relationship between diseases. Additionally, contrary to reality, most models treat the relevance of multimodal information related to disease and drugs as being equal. This paper suggests GCMM to predict potential drug–disease connections using multi-source data in order to overcome all of the aforementioned problems. First, HN are derived from multi-view drug and disease-related information, and the GCN encoder produces drug and disease embeddings based on multi-source similarity. Then, rather than being connected directly, the features are weighted according to the global average pooling of multi-source information attention process. The next stage is a fully connection layer for futher feature learning. Finally, matrix completion is used to determine the drug–disease correlation coefficient for each pair, treating the issue as a recommendation task from an HN. A comparative experiment is also run using four recently proposed deep learning-based models to confirm the validity of the suggested model. It demonstrates that the GCMM outperforms other models in this HN. A case study done on predicting potential treatments for AD further demonstrates the GCMM’s improvement and applicability.

Overall, the main contribution of this paper can be summaried as follows:

- According to study, muti-source of drug and disease information to construst HN is better to extract and fuse information for in silico DR from open-source databases.
- A novel end-to-end GCMM is proposed that can accuratly predict potential relationships and improve performance than four baseline networks. Specifically, analysis of results provides the proof of accuracy and robustness of GCMM.
- Case study conducted on AD indicates GCMM’s availability. Futhermore, 80% of the five drugs with the highest correlation coefficient are supported by previous research and the therapeutic potential of Methicillin on AD is further analyzed.

## Materials and methods

**Fig. 1:**
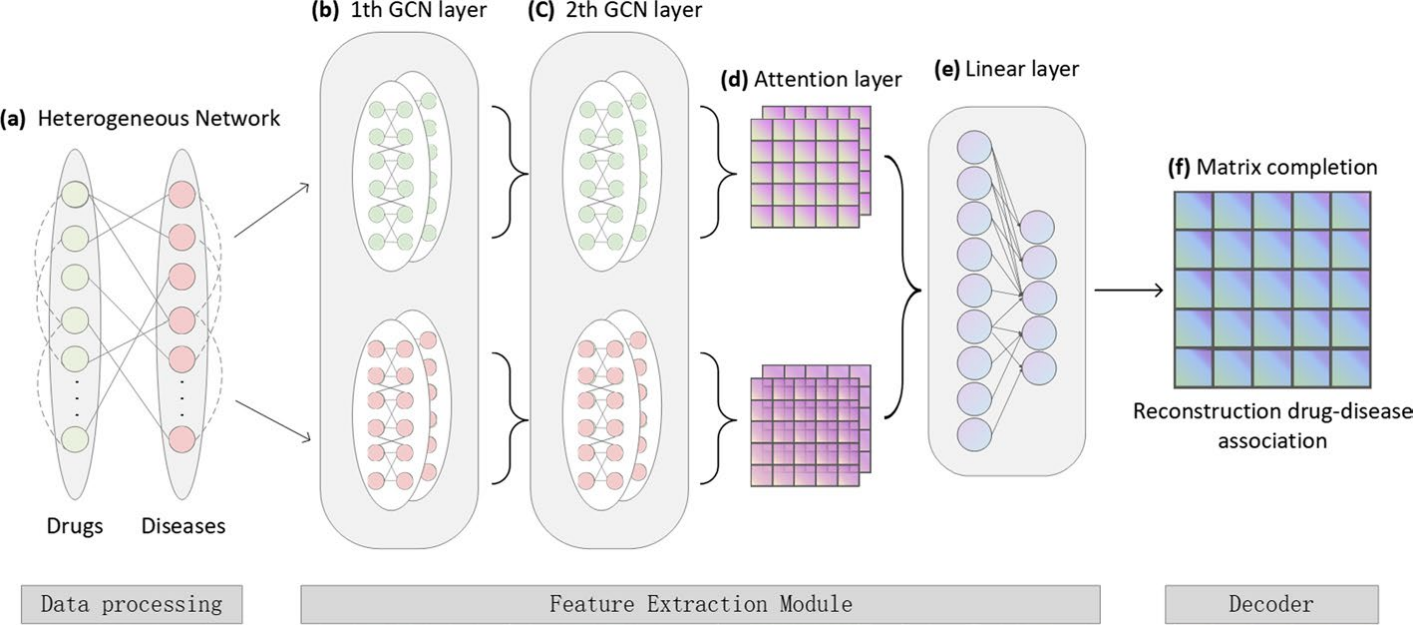
Architecture of GCMM. **a** The construction of HN, which contains multi-source drug and disease information. **b** 1st GCN encoder. It takes HN of drug and disease nodes as input, fuses their neighbor information, and generates embeddings under different views. **c** 2nd GCN encoder. **d** Multichannel attention mechanism on drug and disease. **e** Fully connected feature extractor. **f** Matrix completion decoder

In this paper, the problem of drug–disease prediction is treated as the recommendation task from a HN with drugs, diseases as nodes, and interactions or relationships as edges. As shown in Fig. 1, this section describes the HN constructed from multi-source information, consisting of four kinds of drug–drug, disease–disease similarity, and the experimentally validated drug–disease associations. After that, the workflow of the proposed framework GCMM to predict drug–disease association is illustrated.

### Construction of heterogeneous network

Figure 1a shows the process of building a HN. HN includes the known drug–disease associations, drug–drug chemical similarity $G^C$, drug–drug therapeutic similarity $G^T$, disease–disease semantic similarity $G^M$ and disease–disease target-based similarity $G^A$.

#### The known drug–disease associations

Clinically reported or experimentally verified drug–disease associations from two comprehensive databases are integrated to establish the HN: DrugBank [22] and repoDB [23]. The network includes 5159 experimentally verified drug–disease pairs between 1519 drugs and 728 diseases. The drugs and diseases are normalized through standard terms from Medical Subject Headings (MeSH) [24].

#### Drug–drug chemical similarity

By using Open Babel v2.3.1 [25], Molecular Access System (MACCS) fingerprints [26] can be computed via the SMILES string for the drugs [27]. If two drug molecules $\left( g_i, g_j\right)$ have a and b bits set in their MACCS fragment bit-strings, with c of these bits being set in the fingerprints of both drugs, the chemical similarity [28] $G_{\left( g_i, g_j\right) }^C$ of the drug–drug pair is defined as:

1 $$\begin{aligned} G_{\left( g_i, g_j\right) }^C = \frac{c}{a + b -c} \in \left[ 0,1\right] \end{aligned}$$

$G^C \in R^{N_g \times N_g}$ represents the chemical view of the drug, which $N_g$ indicates the number of drug.

#### Drug–drug therapeutic similarity

Drug therapeutic similarity is measured by the the canonical protein sequences similarity of drug targets, which contains the probability of a therapeutic linkage between drugs. The canonical protein sequences in Homo sapiens is downloaded from Uniprot database (http://www.uniprot.org/). Then the protein sequence similarity $T(e_1, e_2)$ of two drug targets $e_1$ and $e_2$ using the Smith–Waterman algorithm [29]. The Smith–Waterman algorithm performs local sequence alignment by comparing segments of all possible lengths and optimizing the similarity measure for determining similar regions between two strings of protein canonical sequences of drug targets. The overall sequence similarity of the drug targets binding two drugs $g_i$ and $g_j$ is determined by Eq. 2 by averaging all pairs of proteins $e_1$ and $e_2$ with $e_1 \in E_1$ and $e_2 \in E_2$ under the condition $e_1 \ne e_2$.

2 $$\begin{aligned} G_{\left( g_i, g_j\right) }^T = \frac{1}{n_{pairs}}\sum _{e1, e2}T(e_1, e_2) \in \left[ 0 ,1\right] \end{aligned}$$

Matrix $G^T \in R^{N_g \times N_g}$ can be considered as the therapeutic view of the drug.

#### Disease–disease semantic similarity

The National Institute of Health (NIH) database (http://www.ncbi.nlm.nih.gov/) is available for researching the relationship between different diseases. As described in [30], each MeSH representing a disease showed a structure of a hierarchical Directed Acyclic Graph (DAG). For a disease $s_i$, its hierarchical relationship represented by $\mathrm {DAG}(s_i)=\left( {\mathcal {N}}\left( s_i\right) ,\varepsilon \left( s_i\right) \right)$, where ${\mathcal {N}}\left( s_i\right)$ is the set of nodes containing $s_i$ and its ancestors, and $\varepsilon \left( s_i\right)$ denotes the set of direct links from parent nodes to their child nodes. Following previous work [30], diseases that share larger part of their DAGs tend to have higher semantic similarity. The contribution of a node n in $\mathrm {DAG}\left( s_i\right)$ to the semantic value of disease $s_i$ is given by:

3 $$\begin{aligned} F_{s_i}(n) = {\left\{ \begin{array}{ll} 1 &{} \text {if } n = s_i,\\ max\{ F_{s_i}(n') \mid n' \in children\, of\, n\} &{} \text {if } n \ne s_i. \end{array}\right. } \end{aligned}$$

The semantic value of disease $s_i$ is defined as:

4 $$\begin{aligned} DV\left( s_i\right) = \sum _{n \in {\mathcal {N}}}F_{s_i}\left( n\right) \end{aligned}$$

The semantic similarity of two diseases $G_{\left( s_i, s_j\right) }^M$ is defined as:

5 $$\begin{aligned} G_{\left( s_i, s_j\right) }^M = \frac{\sum \nolimits _{n \in {\mathcal {N}}_{\left( s_i\right) } \cap {\mathcal {N}}_{\left( s_j\right) }}\left( F_{s_i}\left( n\right) + F_{s_j}\left( n\right) \right) }{DV\left( s_i\right) + DV\left( s_j\right) }\in [0,1] \end{aligned}$$

$DV\left( s_i\right)$ and $DV\left( s_j\right)$ represents the sematic contribution of disease $s_i$ and disease $s_j$ respectively. Then, the matrix $G^M \in R^{N_s \times N_s}$ symbolizes the sematic view of the disease. $N_s$ is the number of diseases.

#### Disease–disease target-based similarity

Disease target-based similarity measure is measured by using the known drug–disease associations, which contains the probability of a target linkage between diseases. Jaccard similarity algorithm [31] is used to calculate the similarity of nodal structure. $E_i$ and $E_j$ represents target sets that are related to disease $S_i$ and $S_j$ respectively, the target-based similarity $G_{\left( s_i, s_j\right) }^A$ of the disease–disease pair is defined as:

6 $$\begin{aligned} G_{\left( s_i, s_j\right) }^A=\frac{\vert E_i \cap E_j\vert }{\vert E_i \cup E_j\vert }=\frac{\vert E_i \cap E_j\vert }{\vert E_i\vert + \vert E_j\vert -\vert E_i \cap E_j \vert } \in \left[ 0,1\right] \end{aligned}$$

Similarly, matrix $G^A \in R^{N_s \times N_s}$ notes the target-beased view of the disease.

### Model architecture

Based on the HN structure constructed in the previous part, a novel end-to-end graph neural network framework GCMM is proposed to identify the potential drug–disease associations. The model is mainly composed of an encoder and a decoder. To be more specific, as shown in Fig. 1b–f, GCMM consists of the four main modules detailed below: 2-layers multi-view GCN encoder, multimodal based attention mechanism, fully connected feature extractor, and matrix complete decoder.

**Fig. 2:**
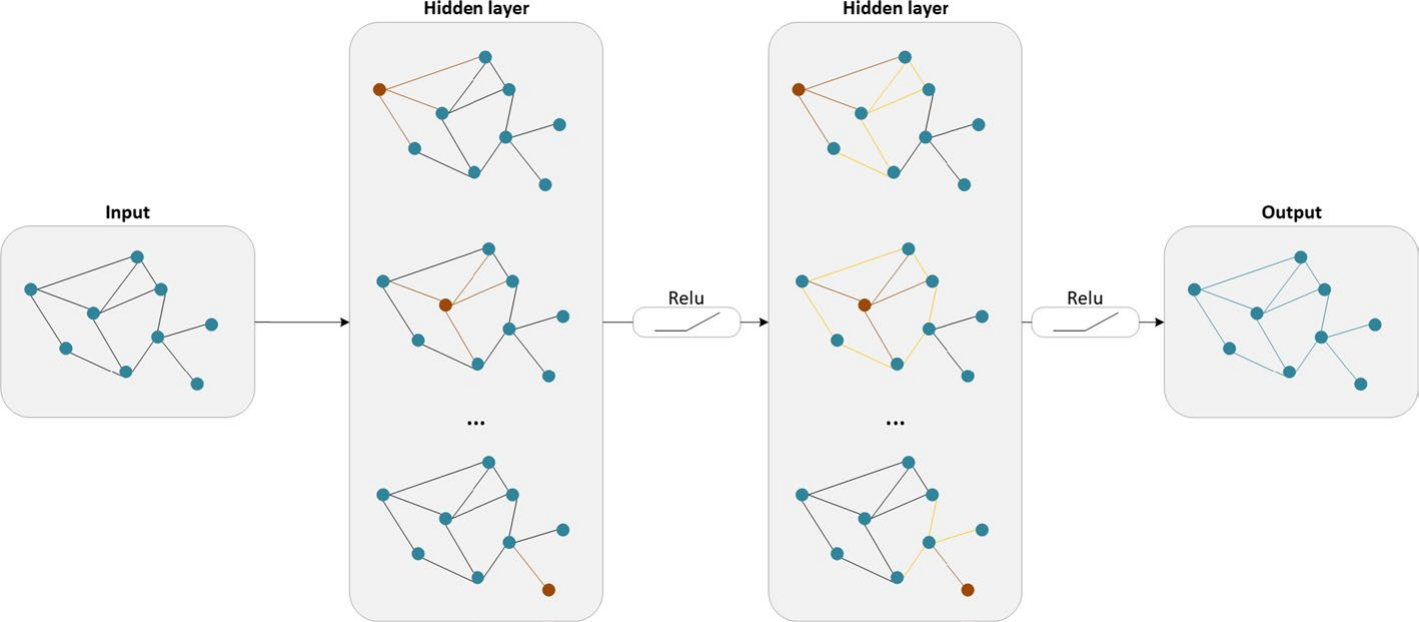
An illustration of GCN encoder

#### Multi-view GCN encoder

Convolutional nerual network(CNN) [32] has been widely used in many fields, such as computer vision, speech recognition, and natural language processing. However, CNN can not be applied to data structures in non-Euclidean space. GCN [33] is a typical spectral model that combines graph convolution and neural networks to achieve the graph task of semi-supervised classification. In particular, GCN uses the Laplacian matrix of a graph to derive its Laplacian operator in the frequency domain, then analogies the convolution in the Euclidean space in the frequency domain to derive the formula of graph convolution. On an application level, GCN and its variants significantly improve many network-related predictive tasks, such as predicting the properties and structure of small biological molecules.

In GCMM, a multi-view GCN encoder on four similarity networks is used to learn drug and disease low-dimensional representations. As Fig. 2 shows, the GCN encoder updates the features by integrating the domain information of nodes in the graph. The learned embeddings are used as input to downstream tasks. Drug nodes embedding can be obtained from the entire graph $G^C$ and $G^T$:

7 $$\begin{aligned} X^{\left( l + 1\right) } = \sigma \left( {\widetilde{D}}^{-\frac{1}{2}}{\widetilde{A}}{\widetilde{D}}^{-\frac{1}{2}}X^{\left( l\right) }W^{\left( l\right) }\right) \end{aligned}$$

where $X^{\left( l + 1\right) } \in R^{N_g \times F_g}$ denotes the $F_g$ dimension features of $N_g$ drugs in $\left( l + 1\right)$th GCN layer. In particular, $X^{\left( 0\right) }$ is randomly initialized and $W^{\left( l\right) }$ is the parameter matrix of model learning. *A* denotes the adjacent matrix for similarity G and the formula is defined as:

8 $$\begin{aligned} {\widetilde{A}} = I + A \end{aligned}$$

$L={\widetilde{D}}^{-\frac{1}{2}}{\widetilde{A}}{\widetilde{D}}^{-\frac{1}{2}}$ is the symmetric normalized Laplacian matrix of G and ${\widetilde{D}}$ is a diagonal matrix with diagonal entry$\left[ {\widetilde{D}}\right] _{ij} = \sum _{j}\left[ {\widetilde{A}}\right] _{ij}$. Analogously, disease nodes feature acquired by similarty graph $G^M$ and $G^A$ as follows:

9 $$\begin{aligned} Y^{\left( l + 1\right) } = \sigma \left( {\widetilde{D}}^{-\frac{1}{2}}{\widetilde{A}}{\widetilde{D}}^{-\frac{1}{2}}Y^{\left( l\right) }W^{\left( l\right) }\right) \end{aligned}$$

Using a multi-layer GCN encoder to the multiple similarity graphs, drug and disease embeddings from different views $\left( X^C, X^T, Y^M, Y^A\right)$ can be obtained.

#### Multimodal based attention mechanism

**Fig. 3:**
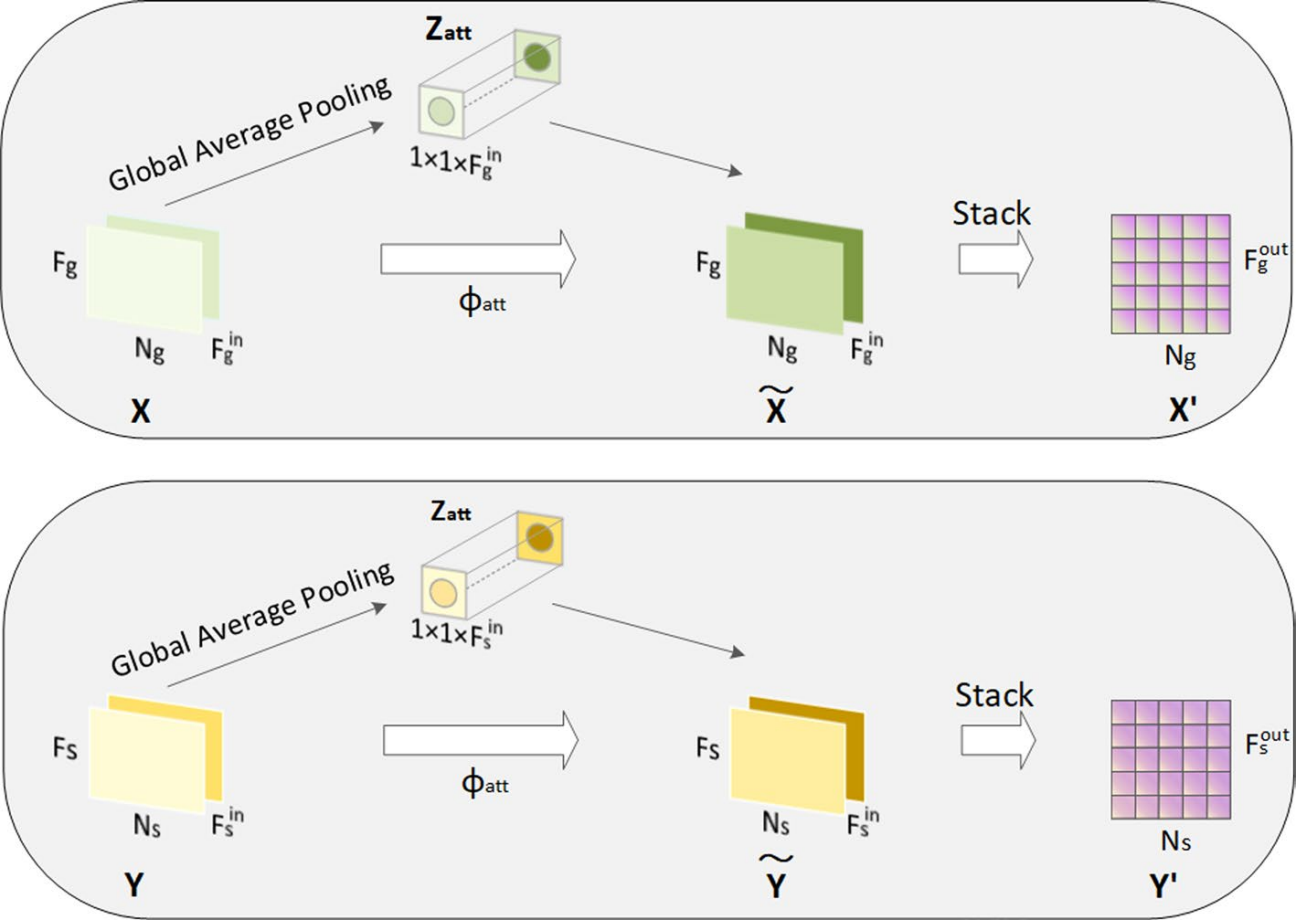
An illustration of Attention layer

Attention mechanism [34] is inspired by the biological system of human that focus on the distinctive parts when processing large amount of information. The model will be more expressive and can hold more data the more parameters it has, but this also introduces the issue of information overload. The issue of information overload can be resolved, and the effectiveness and accuracy of task processing can be enhanced, by introducing attention mechanisms to focus on the information that is more important to the current task, reduce attention to other information, and filter out irrelevant information. Attention has gradually become one of the most important concepts in the deep learning field.

In GCMM, the multimodal-based attention layer is introduced after the multi-view features are obtained. As shown in Fig. 3, it enables the model the ability to distinguish and assigns different weights for multi-source input. Global average pooling is used to calculate the weight of each embedding. For drug with $F_{in}^g$ channels, in this article $F_{in}^g = 2$, its channel statistic $Z_g \in R^{1 \times 1 \times F_{in}^g}$ is calculated by drug’s features $X \in R^{F_g \times N_g \times F_{in}^g}$. For the chemical feature of drug $X^C$, the channel statistic $z_c$ is defined as:

10 $$\begin{aligned} {}{z_c^{att} = \frac{1}{F_g \times N_g}\sum \nolimits _{i = 1}^{F_g}\sum \nolimits _{j = 1}^{N_g}X^C\left( i, j\right) } \end{aligned}$$

And the attention weights of all channels can be computed as:

11 $$\begin{aligned} Z_{att} = \delta \left( W_2\sigma \left( W_1Z_g\right) \right) \end{aligned}$$

where $\delta \left( \cdot {}\right)$ and $\sigma \left( \cdot {}\right)$ represents Sigmoid activation function and Relu activation function, respectively. $W_1$, $W_2$ are the training parameters. Multimodal attention $Z_{att}$ is composed of $Z_{att} = \left[ z_c^{att}, z_t^{att}\right]$. Finally, feature of each view and its corresponding weight coefficient are combined to standardize, for drug in the chemical view and therapeutic view with attention is shown in the 12 and 13 :

12 $$\begin{aligned}&{\widetilde{X}}^{C} = X^C \cdot z_{c}^{att} \end{aligned}$$

13 $$\begin{aligned}&{}{ {\widetilde{X}}^{T} = X^T \cdot z_{t}^{att}} \end{aligned}$$

In the same way, drug and disease attention-based normalized embeddings from different views $\left( {\widetilde{X}}^{C}, {\widetilde{X}}^{T}, {\widetilde{Y}}^{M},{\widetilde{Y}}^{A}\right)$ can be obtained through this module. Drug channel embedding is identified as ${\widetilde{X}} = \left[ {\widetilde{X}}^C, {\widetilde{X}}^T\right]$, disease channel embedding is identified as ${\widetilde{Y}} = \left[ {\widetilde{Y}}^C, {\widetilde{Y}}^T\right]$.

#### Fully connected feature extractor

The fully connected layer is skilled in synthesizing information extracted from the previous section. In this module, it is utilized to integrate multiple view information and generate final embedding. Given drug channel embedding ${\widetilde{X}}= \left[ {\widetilde{X}}^C, {\widetilde{X}}^T\right]$, the final feature $X^{'} \in R^{F_{out}^g \times N_g }$ is defined as:

14 $$\begin{aligned}&Lin_{X} = \sigma (bias + \sum _{i = 1}^{F_{in}^g}{\widetilde{x}} \times W_{X}) \end{aligned}$$

15 $$\begin{aligned}&X^{'} = stack(Lin_{X^C}) \end{aligned}$$

where $W_{X} \in R^{V_g \times 1}$ is the learning parameter, and $Lin_{X} \in R^{1 \times N_g}$ means the output of drug embedding. The final feature of drug $X^{'}$ is computed from stacking the multiple channel outputs. Analogously, disease final embedding $Y^{'}$ can be obtained.

#### Matrix completion decoder

The learned drug and disease embeddings from the encoder are input into the matrix completion module, and the preference prediction problem is treated as a recommendation task. The predicted association matrix $U \in R^{N_g \times N_s}$ is defined as:

16 $$\begin{aligned} U = X^{'T}\cdot Y^{'} \end{aligned}$$

for the values in *U*, $U_{ij}$ is the degree to which drug i is associated with disease j. The goal of GCMM is to minimize the Frebious norm of the difference between *U* and experimentally verified label matrix $U^{'}$. The loss function of the model is defined as follows:

17 $$\begin{aligned} \mathop {argmin}_{\theta }\Vert {U-U^{'}}\Vert ^{2}_{F} \end{aligned}$$

## Results and discussion

### Experiment settings

Known drug–disease association pairs are taken as the positive samples and other pairs as negative instances. Due to the low density of the dataset, 5FCCV is used to evaluate the prediction performance on all positive samples and randomly selected negative instances of the same size. In each round, one subset serves as the valid set and the others as the training set. All experiments are conducted on a single GTX 2080Ti GPU with 11GB of memory on a Linux system. Adam optimization algorithm [35] is used to minimize the loss value druing the model’s training, and 1000 training epochs with the 0.001 learning rate. The area under the receiver operating characteristic (ROC) curve (AUC) and the area under the precision/recall (PR) curve (AUPR) are chosen as the primary evaluation index of robustness. Besides, the threshold-based metrics are also calculated, i.e., Recall (also known as sensitivity), Accuracy(ACC), Precision and F1-measure (F1). The metrics can be calculated by:

18 $$\begin{aligned}&TPR = \frac{TP}{TP + TN} \end{aligned}$$

19 $$\begin{aligned}&FPR = \frac{FP}{TN + FP} \end{aligned}$$

20 $$\begin{aligned}&Precision = \frac{TP}{TP + FP} \end{aligned}$$

21 $$\begin{aligned}&Recall = \frac{TP}{TP + FN} \end{aligned}$$

22 $$\begin{aligned}&ACC = \frac{TP + TN}{TP + TN + FP + FN} \end{aligned}$$

23 $$\begin{aligned}&F1 = 2 \times \frac{Precision \times Recall}{Precision + Recall} \end{aligned}$$

which *TP*, *TN*, *FP*, *FN* means true positive, true negative, false positive and false negative respectively.

### Performance of GCMM on the cross-validation

**Table 1:** Performance of GCMM on 5FCCV

	Auc	Aupr	F1	ACC	Recall	Precision	Specificity
Fold 1	0.8990	0.9110	0.8142	0.8178	0.7984	0.8306	0.8372
Fold 2	0.9000	0.9114	0.8150	0.8130	0.8236	0.8065	0.8023
Fold 3	0.9069	0.9190	0.8168	0.8137	0.8207	0.8043	0.7878
Fold 4	0.9035	0.9135	0.8178	0.8159	0.8266	0.8093	0.8052
Fold 5	0.9002	0.9108	0.8163	0.8169	0.8140	0.8187	0.8198
Mean	**0.9013**	**0.9131**	**0.8160**	**0.8155**	**0.8167**	**0.8139**	**0.8105**

The mean experimental results of 5FCCV are shown in bold font

As shown in Table 1, it is the average of ten experiments. According to the results, it can be observed that GCMM accurately predicts the association between drug and disease and perform robustly in the dataset. The average AUC score is about 0.90, and the average AUPR score is about 0.91. In addition, the deviations of each fold are low, which demonstrates the stability of the model.

### Baseline methods and performance comparison

**Fig. 4:**
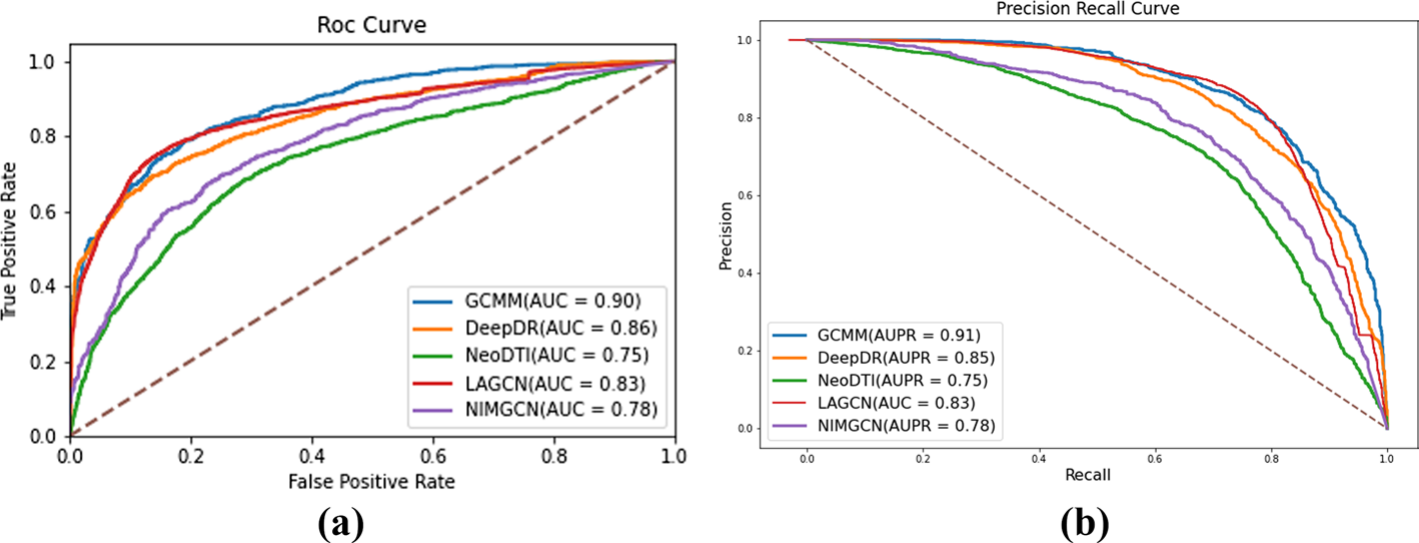
Performance of GCMM and baselines. **a** Validation AUC values of GCMM with other methods. **b** Validation AUPR values of GCMM with other methods

**Table 2:** Performance comparsion on the ratio of positive and negative samples is 1:1

	Auc	Aupr	F1	ACC	Recall	Precision	Specificity
GCMM	**0.9013**	**0.9131**	**0.8160**	**0.8155**	0.8167	**0.8139**	0.8105
DeepDR	0.8574	0.8810	0.7844	0.7705	**0.8174**	0.7539	0.8860
NeoDTI	0.7501	0.7531	0.7149	0.6804	0.8015	0.6452	0.9908
LAGCN	0.8367	0.8470	0.7643	0.7521	0.8100	0.7495	0.9878
NIMGCN	0.7784	0.7807	0.7287	0.7064	0.7888	0.6772	**0.9921**

The mean experimental results of 5FCCV are shown in bold font

**Table 3:** Performance comparsion on all instances

	Auc	Aupr	F1	ACC	Recall	Precision	Specificity
GCMM	**0.8920**	**0.2534**	**0.3266**	**0.9950**	**0.2597**	**0.4401**	0.9984
DeepDR	0.8433	0.2123	0.2864	0.9931	0.2151	0.4332	**0.9990**
NeoDTI	0.7162	0.0717	0.0352	0.9621	0.1094	0.3048	0.9905
LAGCN	0.8270	0.1710	0.1303	0.9921	0.2010	0.4295	0.9818
NIMGCN	0.7587	0.1018	0.0718	0.9874	0.1348	0.3572	0.9818

The mean experimental results of 5FCCV are shown in bold font

Four recently proposed deep-learning models, including DeepDR, NeoDTI, LAGCN, and NIMGCN [18, 20, 21, 36], are chosen as baseline approaches in order to demonstrate the superiority of GCMM’s performance. They are also similarity-based graph neural network models. The training and testing sets of all comparison models are the same as those of GCMM. Training was carried out according to the degree of fit of each model, and the hyperparameters of these models are tuned. First, the same training dataset as GCMM and the ratio of 1:1 positive and negative samples are used to compare these models. The average results of their ten trials are shown in Table 2. Besides, the ROC curve and PR curve are drawn for prediction performance evaluation. As shown in Fig. 4a, ROC curve represents how the true positive rate (TPR) and false positive rate (FPR) change under different thresholds, the model with better classification performance has a larger AUC. As shown in Fig. 4b, The PR curve represents the precision and recall rate changes at different thresholds. The larger the AUPR value is, the better the effect of the model will be. Next, perform a cross-validation test on all pairs, both positive and negative. This scenario basically mimicked the practical situation in which the drug–disease pairs are sparsely labeled. It can be observed that GCMM greatly outperformed other baseline methods, with significant improvement on most indicators from Table 3.

It can be observed that the GCMM model is more optimized than the other models on two primary indexes. Futhermore, other metrics stabilized by GCMM are relatively more stable compared with other methods. The priority of GCMM can be attributed to the following points:

- Graph convolution network has a good effect on feature extraction from similarity graph and fusing heterogeneous information.
- The multi-dimensional attention mechanism is introduced to process multimodal information, especially for the complex drug–disease network.
- The full connection layer can further extract the feature effectively.

### Model ablation experiment

**Table 4:** Performance comparsion between GCMM and its variants

	Auc	Aupr	F1	ACC	Recall	Precision	Specificity
GCMM	**0.9013**	**0.9131**	**0.8160**	**0.8155**	**0.8167**	**0.8139**	**0.8105**
GCMM_no_att	0.8671	0.8793	0.7949	0.7897	0.8149	0.7758	0.7645
GCMM_no_lin	0.8606	0.8526	0.7844	0.7752	0.8178	0.7536	0.7326

The mean experimental results of 5FCCV are shown in bold font

**Fig. 5:**
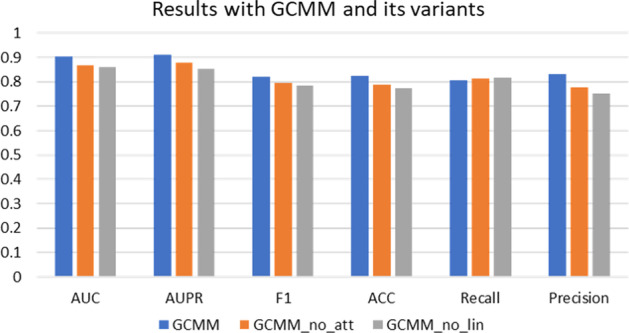
Result with GCMM and its variants

Two GCMM variations are used in the ablation experiment in this section in order to verify the significance of each module in the GCMM.

To determine if a multimodal-based attention layer increases the model’s predictive performance, GCMM without an attention layer (GCMM sans att) is used. The attention mechanism enhances the performance of the GCMM by roughly 3%, as seen in Table 4 and Fig. 5. For GCMM and GCMM without linear layer (GCMM_no_lin), Fig. 5 shows the linear layer futher extract the embedding could improve the metrics by about 4%. The model obtains a high level of prediction accuracy due to the combination of each of its modules.

### The ablation of multi-source information

**Table 5:** Performance of multi-source information

	Auc	Aupr	F1	ACC	Recall	Precision	Specificity
$G^C + G^T + G^M + G^A$	**0.9013**	**0.9131**	**0.8160**	**0.8155**	**0.8167**	**0.8139**	0.8105
$G^C + G^M$	0.8734	0.8891	0.7890	0.7951	0.7665	0.8129	**0.8236**
$G^C + G^A$	0.8685	0.8771	0.7918	0.7844	0.8198	0.7656	0.7490
$G^T + G^M$	0.8432	0.8444	0.7744	0.7452	0.8750	0.6946	0.6153
$G^T + G^A$	0.8657	0.8758	0.7913	0.7912	0.7917	0.7909	0.7907
$G^C + G^T + G^M$	0.8698	0.8781	0.7926	0.7776	0.8491	0.7426	0.7054
$G^C + G^T + G^A$	0.8651	0.8757	0.7900	0.7917	0.7839	0.7963	0.7994
$G^C + G^M + G^A$	0.8827	0.8928	0.8000	0.7955	0.8178	0.7829	0.7733
$G^T + G^M + G^A$	0.8800	0.8908	0.8051	0.8018	0.8188	0.7919	0.7849

The mean experimental results of 5FCCV are shown in bold font

To verify the importance of multi-modal information, the ablation experiments of single and multiple multi-source information are tested.

As shown in Table 5, it is the experimental result of all multi-source information combations. The information of $G^C + G^T + G^M + G^A$ obviously superior to the results of other combations on most metrics. To be specific, its result 3.0% more than the best single information $G^C + G^M$ on AUC and AUPR. Futhermore, its result 2.3% more than the $G^T + G^M + G^A$ on AUC and AUPR.

### Hyper-parameter Analysis

**Fig. 6:**
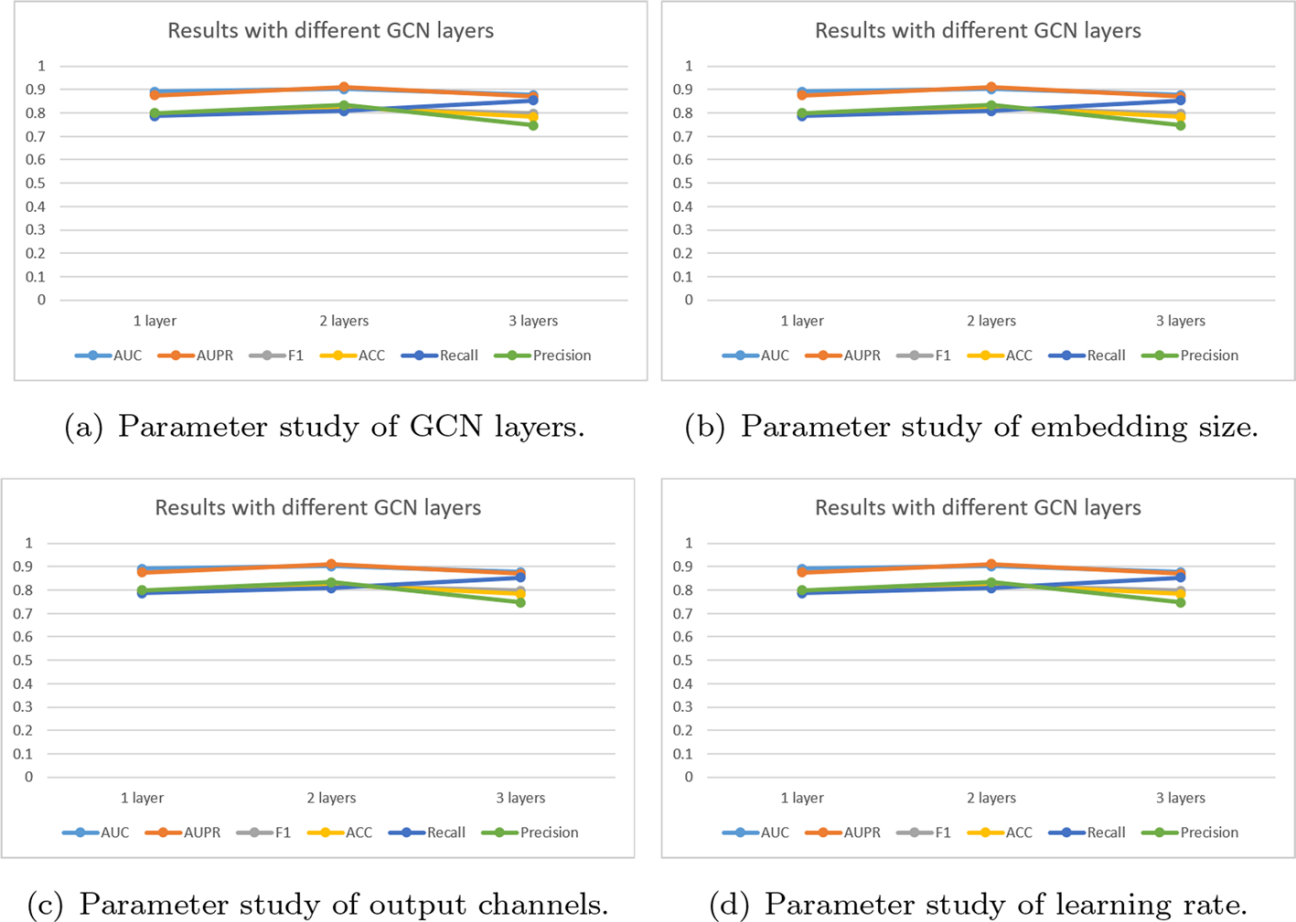
Results of different hyper-parameters

Four important parameters—the number of GCN layers, embedding size, output channels, and learning rate—are examined through experimentation to examine the impact of hyper-parameters on model performance.

- It can be obsevered in Fig. 6a that the 3 layers has the lowest performance, it can be attributed to the limitation of GNNs is the over-smoothing issue [37]. And the result of 1 GCN layer suggests that a shallow GCN can not sufficiently propagate the node feature to fuse heterogeneous information. Meanwhile, it can found that GCMM achieved significant improvement with the appropriate 2 GCN layers.
- The embedding size can directly affect the performance of the GCMM. In the experiment, embedding size is changed in [32, 64, 128, 256, 512] dimensions. From Fig. 6b, within a certain range, the larger embedding size, the higher AUPR and Precision. 256 dimensions is choiced in this thesis.
- Output channel determines the final dimension of the drug and disease features. The output channel is changed in [64, 128, 256]. It can seen from Fig. 6c that AUC and AUPR achieve the highest with 128 output channels in GCMM.
- Learning rate is the degree to which each parameter is optimized as loss function during model training, and its value is related to whether the model can be optimal result. If the learning rate is too high, the parameters to be optimized will fluctuate near the minimum value. On the contrary, too small learning rate will lead to slow convergence of parameters to be optimized. Figure 6d shows the optimum learning rate for the model is 0.001.

## Case study

### New drugs predicted for AD

To further assess the quality of GCMM’s novel prediction, a case study is undertaken using a literature-based evaluation of new drug–disease pairs. Specifically, GCMM is applied to predict candidate drugs for AD. AD is now the most common neurodegenerative disease [38], general dementia is characteristic and the etiology is unknown. The application of drug retargeting as a predictive treatment for AD is of great value.

**Table 6:** New top5 drugs predicted by GCMM for Alzheimer’s disease

	Candidate drug	Evidences
1	Dexamethasone	[39, 40]
2	Cysteamine	[41]
3	Aripiprazole	[42–44]
4	Rifapentine	[45, 46]
5	Meticillin	NA

After calculating the predicted correlations of all drug–disease pairs, a sorted list of top5 drug–disease associations is generated based on the predicted scores. New associations are then obtained by excluding all known drug–disease associations from the dataset. Table 6 shows top5 predicted candidate drugs for AD, and four of them (80%) have literature-reported evidence. Specifically, Dexamethasone ($\left( 11\beta ,16\alpha \right) \hbox {-}9\hbox {-}Fluoro\hbox {-}11$) has the highest predictive correlation coefficient with AD. Dexamethasone levels proved to be an important consideration in AD from [39] and [40] indicates that the combination of acyclovir and Dexamethasone might be an alternative therapy for the treatment of AD. The second is Cysteamine, which is the small molecules the decarboxylated derivative of the amino acid cysteine and a desirable characteristic of drugs targeting neurodegeneration. In [41], Chronic cysteamine treatment resulted in improvements in habituation and spatial learning deficits in the APP-Psen1 mouse model of AD. Thirdly, Aripiprazole is a novel antipsychotic molecule. [42] first compares the efficacy, safety of Aripiprazole with placebo in patients with psychosis associated with AD. [43] futher conducted double-blind experiment for the treatment of psychosis in nursing home patients with AD. [44] finally describes randomized controlled trials evaluating the use of aripiprazole in AD-related psychosis and proved its therapeutic effect. In addition, the fourth molecule Rifapentine (RIF) is an antibiotic used to treat tuberculosis, but prevents curli-dependent adhesion and biofilm formation in E. coli at concentrations below those that affect viability [45]. [46] reports the first direct quantification of RIF from rat brain homogenate, simultaneously studies the clearance of $amyloid\hbox {-}\beta$ and finds that RIF crosses the blood–brain barrier and has a protective effect on AD, and further in vivo studies are under investigation.

### Properties analysis of Meticillin

Since there is no correlation between Meticillin and AD in literature and experimental demonstration, this section analyzes the properties of Meticillin and its similarity to new predictive drugs.

**Fig. 7:**
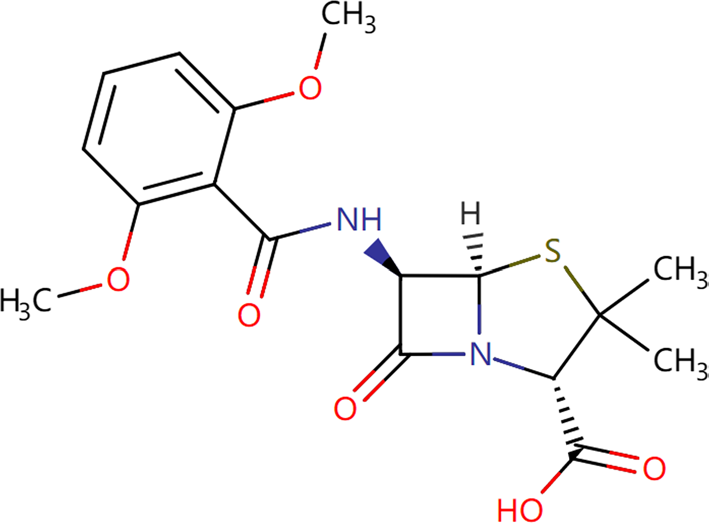
Chemical structure of Meticillin

Methicillin is a penicillin-resistant penicillin, and its antibacterial action is similar to penicillin [47]. Its molecular formula is $C_{17}H_{20}N_2O_6S$ and chemical structure is shown in Fig. 7. Methicillin is mainly used at resistant penicillin staphylococcus caused by all kinds of infection, such as sepsis, respiratory tract infection, meningitis, soft tissue infection, also can be used at pyogenic streptococcus or pneumococcus and resistant penicillin staphylococcus caused by mixed infection [47].

## Conclusion

Drug–disease potential relationships prediction is an important research field of computational drug repurposing to improve drug utilization and guide clinical application. This paper establishes a novel model called GCMM for identifying the potential drug–disease associations. First, GCMM fuses topological information about the similarities of multiple drugs and diseases through the HN by GCN encoders. Second, in contrast to existing methods that assign the same weight to each source, the multimodal attention mechanism is applied to integrate multi-source information. After the full connected layer, the correlation coefficients of each pair of drug–disease are obtained through a matrix completion decoder. Experimental results in 5FCCV demonstrate that GCMM performs better than the other four similarity-based graph neural network models, DeepDR, NeoDTI, LAGCN, and NIMGCN [18, 20, 21, 36], in the majority of indexs, and has a much higher accuracy. In addition, a case study on AD’s potential therapeutic provides specific applications that reaffirms the medical validity of GCMM. All of these results imply the effectiveness and robustness of GCMM and supported by the finding the novel predicted drug–disease associations for drug repurposing. In future research, it is a worthwhile area to examine how to increase the dependability and diversity of biological information with the low sparsity of biological data. Morever, additional biological components, including as proteins, miRNAs, and biological processes, that are implicated in the medication treatment of diseases can be added to the HN.

## Data Availability

The datasets generated and/or analysed during the current study are available in the Github repository, https://github.com/FanZhang0820/GCMM
